# Molecular Perspectives of Mitochondrial Adaptations and Their Role in Cardiac Proteostasis

**DOI:** 10.3389/fphys.2020.01054

**Published:** 2020-08-27

**Authors:** Shafiul Alam, Chowdhury S. Abdullah, Richa Aishwarya, Mahboob Morshed, Md. Shenuarin Bhuiyan

**Affiliations:** ^1^Department of Pathology and Translational Pathobiology, Louisiana State University Health Sciences Center, Shreveport, LA, United States; ^2^Department of Molecular and Cellular Physiology, Louisiana State University Health Sciences Center, Shreveport, LA, United States

**Keywords:** mitochondria, cardiac proteostasis, mitochondrial proteostasis, mitochondrial dysfunction, mitochondrial unfolded protein response, proteotoxicity

## Abstract

Mitochondria are the key to properly functioning energy generation in the metabolically demanding cardiomyocytes and thus essential to healthy heart contractility on a beat-to-beat basis. Mitochondria being the central organelle for cellular metabolism and signaling in the heart, its dysfunction leads to cardiovascular disease. The healthy mitochondrial functioning critical to maintaining cardiomyocyte viability and contractility is accomplished by adaptive changes in the dynamics, biogenesis, and degradation of the mitochondria to ensure cellular proteostasis. Recent compelling evidence suggests that the classical protein quality control system in cardiomyocytes is also under constant mitochondrial control, either directly or indirectly. Impairment of cytosolic protein quality control may affect the position of the mitochondria in relation to other organelles, as well as mitochondrial morphology and function, and could also activate mitochondrial proteostasis. Despite a growing interest in the mitochondrial quality control system, very little information is available about the molecular function of mitochondria in cardiac proteostasis. In this review, we bring together current understanding of the adaptations and role of the mitochondria in cardiac proteostasis and describe the adaptive/maladaptive changes observed in the mitochondrial network required to maintain proteomic integrity. We also highlight the key mitochondrial signaling pathways activated in response to proteotoxic stress as a cellular mechanism to protect the heart from proteotoxicity. A deeper understanding of the molecular mechanisms of mitochondrial adaptations and their role in cardiac proteostasis will help to develop future therapeutics to protect the heart from cardiovascular diseases.

## Introduction

Proteins are complex macromolecules with versatile molecular functions implicated in every biological process (Hartl et al., 2011). The precise folding of newly synthesized proteins is essential for proper functioning through numerous posttranslation modifications. Protein quality control (PQC), the mechanism controlling protein homeostasis, includes protein maturation, transportation to the executive site, and degradation of misfolded or overproduced proteins. Protein homeostasis plays a crucial role in the maintenance of overall cellular health, and its alteration results in the activation of either adaptive signaling pathways to avoid pathological consequences or maladaptive pathways that lead to cellular death (Kroemer et al., 2009; Galluzzi et al., 2015). The term “proteotoxicity” refers to the cellular pathogenic features that result from protein misfolding and aggregation, which is regulated by myriad factors that affect protein folding and misfolding. Under conditions of environmental stress or disease, protein folding is disturbed, which leads ultimately to the generation of misfolded intermediates. Protein homeostasis in cardiomyocytes is subject to several layers of surveillance by the PQC system. Early in the process, misfolded proteins are refolded or directed to degradation by the molecular chaperones. If refolding is impossible due to the extent of misfolding and/or the size of the protein, the ubiquitin-proteosome system or autophagy (including mitochondrial autophagy, mitophagy) ensures protein degradation as a secondary defense mechanism. Some pathological conditions result in the PQC system becoming so overwhelmed that misfolded proteins accumulate, resulting in aggregate formation (Fauconnier, 2018). In addition to these classical PQC system processes, mitochondria play an important role in cytosolic proteostasis through the uptake of aggregates by the mitochondrial import system, which degrades them using mitochondrial serine proteases (Ruan et al., 2017).

The heart is a terminally differentiated organ that undergoes adaptive remodeling by changing cardiomyocyte size as well as mitochondrial content to help it cope with pathological stress (Fauconnier, 2018). The extent of changes in the activity or inhibition of the activity of the PQC system causes one of two results for cardiomyocytes: hyperactivity of the PQC system, leading to the excessive degradation of proteins and thus myolysis, or inactivation of the PQC system, leading to cardiac failures such as amyloidogenic cardiomyopathy, idiopathic cardiomyopathy, hypertrophic cardiomyopathy, dilated cardiomyopathy, or atrial fibrillation. The observed mitochondrial dysfunction related to these pathologies includes alteration of mitophagy, aberrant mitochondrial fission/fusion, or altered branched-chain amino acid catabolism (Huang et al., 2011; Nan et al., 2017). Currently, the mechanisms underlying mitochondrial dysfunction that contribute to the initiation or acceleration of cardiac proteostasis remain elusive. This review encompasses current understanding of the molecular insights concerning the adaptive changes in mitochondria in cardiac proteostasis, the maladaptive effect of mitochondrial dysfunction in cardiac proteostasis, and the impact of impaired cardiac proteostasis on mitochondrial function.

## Challenges of Proteostasis in the Heart

Cardiomyocytes are highly contractile cells requiring a large amount of energy to maintain contractility on a beat-to-beat basis. In the heart, mitochondria occupy nearly one-third of the cardiomyocyte volume (Zhou and Tian, 2018). Due to their high contractility and abundance of mitochondria, cardiomyocytes are subject to continuous generation of proteotoxic agents and the cellular stress response. Cardiomyocytes, as terminally differentiated cells, confront several extra obstacles in their efforts to maintain cellular proteostasis compared to most other cells. Firstly, cardiomyocytes contain highly specialized proteins that participate in electrical conduction and contraction. Therefore, these demand specialized PQC surveillance, such as that provided by small heat shock proteins [e.g., αB-crystallin (CryAB)] that favorably bind to the proteins of the sarcomere to preserve healthy cardiomyocyte function (Golenhofen et al., 2000). Secondly, cardiomyocytes abundantly express heat shock proteins (e.g., heat shock protein 70 (HSP70) that work in both the cytosol and in the mitochondria to maintain mitochondrial proteostasis. In addition, mitochondria in cardiomyocytes have their own PQC system comprising abundant proteases such as Lon Protease I (LonP1), metalloendopeptidase OMA1 (OMA1), and caseinolytic mitochondrial matrix protease proteolytic subunit (ClpP) (Acin-Perez et al., 2018; Smyrnias et al., 2019; Venkatesh et al., 2019). Thirdly, cardiomyocytes are terminally differentiated cells, making them unable to clear protein aggregates during cell division. The protein aggregates accumulate at the microtubule-organizing center in dividing cells during mitosis; one daughter cell receives all of the aggregates through asymmetric distribution, leaving the other one free of aggregates. The daughter cell that contains the asymmetrically distributed aggregates undergoes apoptosis (Rujano et al., 2006). Therefore, cardiomyocytes depend greatly on a balanced PQC system to counteract stress-induced protein misfolding and aggregation (Powers and Balch, 2013; Willis and Patterson, 2013). Fourthly, the huge metabolic demands made by continuously contracting cardiomyocytes are met by oxidative phosphorylation in the mitochondria, which also generates highly reactive and abundant proteotoxic reactive oxygen species (ROS) as a by-product even under healthy conditions (Zhang et al., 2013). A growing number of studies suggest that intricate cross-talk occurs between mitochondrial quality control and cellular proteostasis in cardiomyocytes under pathophysiological conditions.

## Mitochondrial Position, Distribution, and Communication in Cardiac Proteostasis

Healthy mitochondrial function is affected by proper mitochondrial positioning with respect to other organelles, as well as their communication and distribution. Mitochondria show substantial plasticity in shape and distribution and display dynamic behavior in positioning in growing cells. Mitochondria in cardiac muscle are often confined to specific cytoplasmic regions rather than being randomly distributed (Rappaport et al., 1998; Yaffe, 1999). Numerous studies in both animal models and isolated cell cultures have suggested that proper mitochondrial function relies on the interaction among the cytoskeleton, the mitochondria, and other organelles. In the healthy heart, desmin upholds the proper mitochondrial positioning along the sarcomere and plays a crucial role in maintaining normal mitochondrial function by preserving mitochondrial spatial organization (Bär et al., 2004). Desmin intermediate filaments (IF) in skeletal and cardiac muscle connecting the interfibrillar space between neighboring Z discs establish a connection between mitochondria and the IF cytoskeleton (Tokuyasu et al., 1983). Desmin disorganization can alter mitochondrial positioning, compromise mitochondrial function, and lead to cardiomyocyte dysfunction (Bär et al., 2004). The molecular function of desmin networks in maintaining mitochondrial position along with other organelles in cardiomyocytes was revealed in studies using desmin−null mice. These mice displayed a progressive and generalized myopathy affecting the function and structure of the myocardium. The striated muscles in desmin-null mice showed irregular mitochondrial shape and distribution, with a hallmark aggregation of the sarcolemmal mitochondria attributable to weakened muscles and increased fatigue (Li et al., 1996; Milner et al., 1996).

Clinically relevant mouse models of cardiac proteotoxicity, such as the mutant desmin transgenic mouse with 7-amino acid deletion R172-E178 in desmin (D7-Des Tg) (Goldfarb et al., 1998; Dalakas et al., 2000; Wang et al., 2001; Zheng et al., 2011), display a collapse of the desmin network and an accumulation of desmin aggregates that contributes to the development of cardiomyopathy (Wang et al., 2001). Collapse of the desmin network results in early perturbations in mitochondrial structure. We recently reported that the D7-Des Tg mouse model of desmin-related cardiomyopathy (DRC) demonstrated altered mitochondrial morphology and localization along the sarcomere resulting in mitochondrial dysfunction (Alam et al., 2018). Disorganization and collapse of the desmin network were also observed in another mouse model of DRC developed by cardiomyocyte-specific overexpression of mutant (R120G) CryAB (CryAB^R120G^). The chaperone protein CryAB is a small heat shock protein responsible for the maintenance of the desmin network. Mutation of CryAB^R120G^ perturbed the desmin network leading to the disruption of the sarcomere structure (Maloyan et al., 2005). These collapses in desmin networks contribute to altered mitochondrial position and distribution, resulting in dysfunctional cardiac proteostasis and the development of cardiomyopathy.

Microtubulin (MT) plays an important role in the maintenance of sarcoplasmic/ER-mitochondrial contact; disruption of this contact is attributed to the alteration of mitochondrial position (Friedman et al., 2010, 2011; Lewis et al., 2016; Phillips and Voeltz, 2016). Acetylation of the tubulin leads to the stabilization of MT playing a pivotal role in maintaining ER-mitochondria contact. Increased activity of histone deacetylase 6 (HDAC6) induced depolymerization, which perturbed the contact site (Friedman et al., 2010). Indeed, increased activity of HDAC6 leading to mitochondrial-ER contract site disruption has been reported in atrial fibrillation (AF) (Zhang et al., 2014). Muscle mitochondria are confined to a specific subcellular domain within sarcomeres (Stromer and Bendayan, 1988, 1990; Ogata and Yamasaki, 1997), suggesting immobilization of the mitochondria through stable linkages within the cytoskeleton. However, several studies have suggested that mitochondria may also undergo translocation in muscles. Active transport of mitochondria along MT occurs in the axon of neurons through the involvement of kinesin (Leopold et al., 1992; Jellali et al., 1994; Nangaku et al., 1994; Elluru et al., 1995). Some studies have also provided evidence of conventional MT-dependent molecular motor kinesis in the heart and involvement of the kinesin associated with cardiac mitochondria in the dynamic interaction between the organelle and the MT (Lindén et al., 2001). However, the association between kinesin and the mitochondria is absent in the desmin-null mouse heart tissue, suggesting a possible role for intermediate filament (IF) organization in kinesin-mediated interactions between MT and mitochondria (Lindén et al., 2001). Interestingly, new evidence has suggested that membrane nanotubes (MNT) mediated the transportation of cardiac mitochondria between cardiomyocytes and fibroblasts. MNT are identified as long, thin, membrane-based distant connections (Shen et al., 2018) requiring MT and motor protein kinesin for the movement of mitochondria. MNT are also known to be involved in the intercellular transfer of calcium (Ca^2+^) to the mitochondria (He et al., 2011). For the maintenance of proper cardiac function, intimate communication between cardiomyocytes and cardiac fibroblasts is very important (Baudino et al., 2008; Porter and Turner, 2009). Although *in vitro* coculture of neonatal rat cardiomyocytes and fibroblasts has suggested the involvement of the MNT-like structure, there is no direct evidence of mitochondrial transport through the MTN *in vivo* in the adult heart, where the mitochondria are confined within a rigid structure. Despite considerable debate regarding mitochondrial transport in the heart, we can speculate that the disorganization of the desmin network in cardiac proteotoxicity perturbs the transport of mitochondria between cardiomyocytes and fibroblasts, affecting normal cardiomyocyte function.

Recent studies have also suggested that mitochondrial intercommunication through the nano-tunnel is necessary to maintain cellular proteostasis. For the maintenance of mitochondrial function, exchanges of mitochondrial matrix contents between mitochondria are essential. Cardiac mitochondrial matrix exchanges can occur either through direct contact with adjacent mitochondria, called mitochondrial kissing, or by extrusion of a tubular protrusion, called a nano-tunnel, for communication with distant mitochondria. Mitochondrial kissing, the result of physical contact between two adjacent mitochondria, allows transient and incomplete mixing without affecting the mitochondrial membrane potential. Use of a nano-tunnel, a dynamic thin tubular protrusion bridging mitochondria at a relatively long distance, allows the continuous and complete mixing of mitochondrial content without affecting the intermediate mitochondria (Huang et al., 2013). It has been well established that mitochondrial Rho GTPase 2 (Miro2) in the neuronal system plays an important role in the transport of mitochondria to the site of energy demands (Nguyen et al., 2014). Although mitochondrial transportation is believed to be difficult in cardiomyocytes due to their rigid sarcomeric structure, the abundance of Miro2 in cardiomyocytes suggests their possible direct function in mitochondrial intercommunication (Cao et al., 2019). This possibility is supported by a study showing that adenovirus-mediated expression of Miro2 in cardiomyocytes increased the inter-mitochondrial communication through both adjacent mitochondrial kissing and nano-tunneling between distant mitochondria. This finding suggests that in adult cardiomyocytes, Miro2 may be involved in mitochondrial intercommunication through mitochondrial kissing and nano-tunneling, rather than mitochondrial transportation. Interestingly, Miro2 transgenic mice showed increased inter-mitochondrial communication, improved mitochondrial function, and ameliorated cardiac function in a transverse aortic constriction (TAC) model of cardiac injury (Cao et al., 2019). Therefore, these studies suggest that mitochondrial inter-communication plays a critical role in preserving cardiac structure and function.

## Electron Transport Chain and Oxidative Stress in Cardiac Proteostasis

**FIGURE 1:**
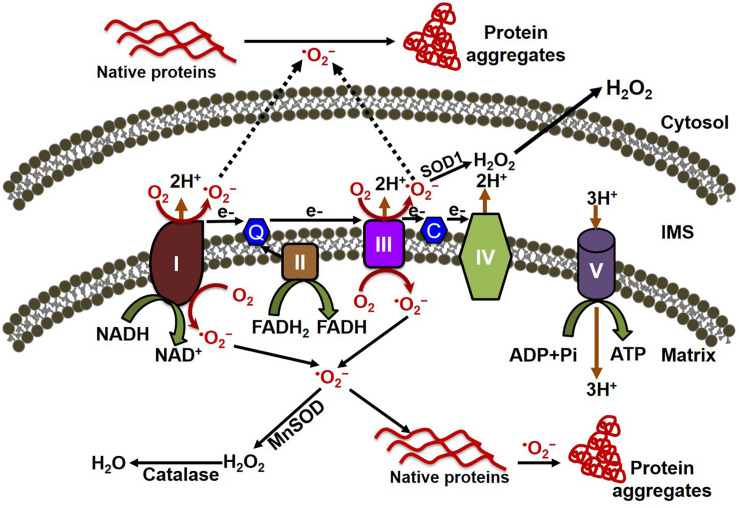
Mitochondrial oxidative stress contributes to both cytosolic and mitochondrial protein aggregation. The electron transport chain (ETC) of the mitochondria, located in the inner mitochondrial membrane is composed of five multi-subunit enzyme complexes (denoted by I, II, III, IV, and V). Electron (e-) flow through the ETC follows the order of electrons donated by coenzymes (NADH and FADH_2_) as they are accepted and transferred to complex I (NADH ubiquinone reductase) or complex II (succinate dehydrogenase) and then consecutively to complex III (ubiquinol-cytochrome C reductase), complex IV (cytochrome c oxidase), and finally to oxygen (O_2_) to produce water (H_2_O). The electrochemical gradient established through the electron transfer along with the ETC couple protons transport across the inner membrane, resulting in ATP generation through complex V (F0F1 ATP synthase). Electron leakage in the ETC, mainly from complex I and III, generates reactive oxygen species such as superoxide anion (O_2_^–^). The transfer of electrons to O_2_ generates O_2_^–^, which is then converted to hydrogen peroxide (H_2_O_2_) by the enzymes superoxide dismutase (MnSOD) and catalase in the mitochondria. O_2_^–^ can oxidize either the mitochondrial protein inside the mitochondria, forming the misfolded protein aggregate that affects the mitochondrial function, or the cytosolic protein after being released into the cytosol, leading to the formation of protein aggregate in the cytosol.

ROS have been implicated in the pathophysiology of cardiovascular diseases and aging processes (Munzel et al., 2017). Studies have shown the involvement of ROS in several cardiac pathophysiologies, such as hindrance in excitation–contraction coupling (Zhang et al., 2015), induction of arrhythmias (Wagner et al., 2014; Kim et al., 2017), and cardiac hypertrophy (Ago et al., 2008). ROS are formed as by-products of cellular respiration during energy production and metabolism, and also by some specialized enzymes (Burgoyne et al., 2012). Mitochondria are the major source of ROS production, along with their primary functions involved with energy production and metabolism (Munzel et al., 2015). The mitochondrial electron transport chain (ETC) is composed of flavoprotein-containing complexes and super-complexes (Letts et al., 2016). During electron transport and O_2_ reduction (respiration), small numbers of electrons can move from complexes I and III to form superoxide (⋅O_2_^–^), which in turn is converted to the more stable hydrogen peroxide (H_2_O_2_) by manganese (Mn)-dependent superoxide dismutase (MnSOD) (Balaban et al., 2005). Although ROS intermediate products (e.g., ⋅O_2_^–^) and hydroxyl radicals (⋅OH) have extremely short half-lives (milliseconds to nanoseconds), they are capable of oxidizing and damaging almost all organic molecules (Leichert and Dick, 2015; Figure 1). More stable ROS (e.g., H_2_O_2_) produced during the detoxification of ⋅O_2_^–^ can serve as mediators of intracellular signaling. Maintaining optimal levels of ROS balance through production and detoxification is critical for proper cellular function and survival (Aon et al., 2010). Mitochondrial ETC complexes also possess an iron–sulfur cluster (Chang et al., 2016), important for the function of the oxidation–reduction reactions of mitochondrial electron transport. Multiple iron–sulfur clusters have been reported in both Complex I and Complex II of oxidative phosphorylation. ROS have been shown to affect the stability and function of the iron–sulfur cluster (Jang and Imlay, 2007). In the degenerative disease Friedreich’s ataxia, ROS have been shown to damage mitochondrial iron–sulfur clusters, triggering heme deficiency, and leading to further generation of ROS (Napoli et al., 2006). However, a direct link between the altered iron–sulfur cluster and cellular proteostasis remains unknown.

Under normal physiological conditions, the increased levels of ROS are involved in pro-survival signaling (Zorov et al., 2014). The sensitivity of mitochondria to ROS within the heart varies due to the heterogenicity of the spatial mitochondrial distribution. While the majority of the mitochondria are situated between myofibrils (interfibrillar mitochondria, IMF), the rest are located beneath the sarcolemma (subsarcolemmal mitochondria, SSM) and around the nucleus (perinuclear mitochondria, PNM) (Lu X. et al., 2019). Therefore, mitochondria have a varying capacity for oxidative phosphorylation depending upon their spatial distribution, as demonstrated in data from a study showing IFM with a 50% higher level of substrate oxidation, as well as higher activity of oxidative phosphorylating enzymes, compared to SSM (Shimada et al., 1984). Moreover, data from a recent study have shown that, compared to PNM, IFM exhibited a greater sensitivity to oxidative stress (Lu X. et al., 2019). It is plausible that IFM could generate more ROS, as increased mitochondrial respiration is associated with an increase in ROS production due to increased ETC activity (Zhou and Tian, 2018). The majority of the cardiac mitochondria are IFM to meet the high energy demands of myofibrillar contractility. Taken together, the data from these studies lead to the speculation that, under pathological conditions, cardiac mitochondria are more susceptible to oxidative stress than non-cardiac dividing cells; however, direct evidence from comparative studies is still lacking.

In the heart, MnSOD plays a critical role to counteract ROS. MnSOD is encoded by the nuclear genome but localizes in the mitochondria through the mitochondrial targeting sequence. MnSOD represents 90% of the activity in the cardiomyocytes. The rest of the SOD is primarily copper-zinc (CuZn) SOD (CuZnSOD or SOD1), which resides mainly in the cytosol. Extracellular SODs are encoded by distinct genes but are also CuZn-containing enzymes (Assem et al., 1997). As MnSOD is located in the mitochondria, representing 90% of SOD activity in cardiomyocytes, it plays a vital role by counteracting oxidative stress in the mitochondria. MnSOD dysfunction causes severe oxidative stress in the mitochondria as well as at the cellular level. Oxidative stress can cause posttranslational modification of the protein, which ultimately alters its function. When under oxidative stress, the amino acids of cardiac proteins undergo modification, reversibly or irreversibly; the extent of the modification depends on the severity of the reactive nitrogen and oxygen species (RNOS) exposure (Kumar et al., 2012; Chung et al., 2013; Go and Jones, 2013). Irreversible oxidative stress-induced posttranslational modification can initiate the pathway involved in protein degradation as well as causing deregulation of protein folding and impairing the clearance of misfolded proteins (Gregersen et al., 2005). Highly oxidized aggregates are resistant to the proteolytic system due to their bulky size and can block the proteasome, leading to the inhibition of proteasomal function (Ayyadevara et al., 2015, 2016). Oxidized lipofuscin aggregate has been reported in aging (Kakimoto et al., 2019) and also neurodegenerative proteotoxic disease (Hohn et al., 2011; Kakimoto et al., 2019). The generation of ROS by damaged mitochondria leads to the oxidation of lipofuscin, inhibiting proteasome activity and resulting in aggregate accumulation (Kakimoto et al., 2019). Moreover, studies have shown a positive correlation between intracellular lipofuscin contents and ROS production and mitochondrial damage in primary cardiomyocytes (Terman et al., 2004) and also in HeLa cells (Konig et al., 2017). Moreover, lipofuscin accumulation was also observed in human cardiac samples obtained from sudden cardiac death (Kakimoto et al., 2019), the end stage of human heart failure with dilated cardiomyopathy and ischemic cardiomyopathy (Rayment et al., 1999; Radu et al., 2012; Nozynski et al., 2013). Lipofuscin accumulation in cardiac proteotoxicity indicates that mitochondrial oxidative stress plays a causative role in both disease initiation and progression.

Similarly, oxidative stress has also been implicated in the cardiac proteotoxicity induced by amyloidosis. Particularly, amyloidogenic light chain (LC) proteins from human patients with amyloid cardiomyopathy affect cellular redox, cause increased ROS production, and lead to impaired contractility and relaxation in cardiomyocytes (Brenner et al., 2004). The mechanisms of increased cellular ROS production may be due to the mitochondrial dysfunction induced by proteotoxicity, similar to that observed in neurodegenerative diseases (Miranda et al., 2000; Eckert et al., 2003). Similarly, human primary cardiac fibroblasts exposed to amyloidogenic LC isolated from light chain amyloidosis patients also induced oxidative stress in the mitochondria (Imperlini et al., 2017).

## Mitochondrial Gene Mutations in Cardiac Proteostasis

Several pieces of evidence indicate that mutation in the genes encoding for mitochondrial ETC complex proteins may affect cellular proteostasis. Mutations in mitochondrial complexes I, II, and IV components are associated with Leigh syndrome, a neurological disorder; they have also been associated with hypertrophic or dilated cardiomyopathy (Berardo et al., 2011). Similarly, a recessive homozygous mutation in succinate dehydrogenase (SDH), a TCA cycle enzyme involved in linking electron transfer from the TCA cycle to mitochondrial complex II, has been shown to lead to the development of prenatal hypertrophic cardiomyopathy with severe complex II deficiency (Alston et al., 2015). Microdeletion of mitochondrial complex III component cytochrome B has also been linked to multisystem disease including left ventricular hypertrophy in humans (Carossa et al., 2014). Mutations in the complex IV components cytochrome C oxidase assembly homolog 10 (COX10) and 15 (COX15) have been shown to have a relationship with hypertrophic cardiomyopathy (Antonicka et al., 2003a, b). COX10 and COX15 are assembly factors of complex IV and play a critical role in the mitochondrial heme biosynthetic pathway by catalyzing the conversion of protoheme (heme B) to heme A (Antonicka et al., 2003a, b). Mutation in the cytochrome oxidase assembly factor 6 homolog (COA6) has been associated with hypertrophic cardiomyopathy with severe complex IV deficiency, and a mild decrease in complex I activity in heart tissue (Calvo et al., 2012; Baertling et al., 2015). COA6 is involved in the stability of the cytochrome C oxidase subunit 2 (COX2); mutation of COA6 results in COX2 deficiency in the heart (Pacheu-Grau et al., 2015, 2018). Mutation during the synthesis of cytochrome oxidase1 (SCO1) and 2 (SCO2) has been reported to lead to the development of hypertrophic cardiomyopathy (Jaksch et al., 2000; Stiburek et al., 2009; Pacheu-Grau et al., 2015). SCO1 and SCO2 are responsible for the transfer of copper to COX1 and COX2 (Herrmann and Funes, 2005; Pacheu-Grau et al., 2015). In general, mutation in these proteins leads to impairment of mitochondrial respiration and ATP production and enhanced ROS production and ultimately causes the cellular stress that impairs bioenergetics. The direct manner by which mutations in mtDNA alter mitochondrial quality control has been reported in a recent study using the mtDNA mutator mouse, which expresses a proofreading-deficient (D257A) version of mitochondrial polymerase gamma (PolG) (Joseph et al., 2013). mtDNA mutations in the PolG mouse altered several mitochondrial quality-control processes, including biogenesis, fusion/fission, and autophagy, contributing to the development of sarcopenia (Joseph et al., 2013). In fact, a wide range of aged tissues from both humans and animals demonstrated increased levels of mtDNA point mutations and deletions linked to a number of pathological conditions (Corral-Debrinski et al., 1992; Wallace, 2001; Wanagat et al., 2002; Khaidakov et al., 2003). Moreover, the loss of muscle mass observed in PolG mice is closely associated with reduced ETC complexes, impaired mitochondrial bioenergetics, and induction of apoptosis (Kujoth et al., 2005). Despite all of these studies linking mtDNA mutations to altered mitochondrial quality control, it remains unknown whether they affect cellular proteostasis, turnover of the whole organelle, or both processes.

## Mitochondrial Ca^2+^ Susceptibility and Cardiac Proteostasis

Cardiomyocytes possess a unique feature: a large and variable intracellular calcium (iCa^2+^) flux that regulates myocyte contraction on a beat-to-beat basis (Bers, 2008). The Ca^2+^ environment in the heart is dynamic to cope with this variable flux of Ca^2+^. Moreover, the demands of the heart force the cardiac mitochondria to have an intricate and super-regulated exchange system competent to deal with these variable changes in Ca^2+^ load. Ca^2+^ influx to the mitochondrial matrix occurs via the mitochondria calcium uniporter (MCU). This influx is counteracted by efflux, which occurs through the mitochondrial sodium–calcium exchanger (NCLX) (Luongo et al., 2017). The mitochondrial membrane potential (Δψ = approximately –180 mV) generated by the proton gradient across the electron transport chain drives the MCU to take up Ca^2+^ (Kirichok et al., 2004). Mitochondria, the main site of the oxidative metabolism that generates ATP, are tightly controlled by the intra-mitochondrial Ca^2+^ concentration, which is closely aligned with the cellular metabolic demand (Berg et al., 2002). Several studies have demonstrated a correlation between an increased mitochondrial Ca^2+^ (mCa^2+^) load and an increase in oxidative phosphorylation and ATP production (Unitt et al., 1989; Brandes and Bers, 2002). Therefore, Ca^2+^ appears to modulate mitochondrial metabolism via various mechanisms, including the regulation of Ca^2+^-dependent dehydrogenases and modulation of ETC complexes (Glancy and Balaban, 2012). Despite the critical role played by Ca^2+^ in meeting the energy demands of cardiomyocytes, numerous studies have also demonstrated the detrimental effects of mCa^2+^ overload on the cardiomyocyte through activation of apoptosis and necrosis (Rasola and Bernardi, 2011). Ca^2+^ is also implicated as the major priming agent in the opening of the mitochondrial permeability transition pore (MPTP), resulting in the collapse of Δψ and dampening of ATP production, which activates apoptotic and necrotic cell death (Foo et al., 2005).

Recently, using the DRC mouse model, we demonstrated that mitochondrial MPTP opening is critical to the development of mitochondrial dysfunction in the heart (Alam et al., 2018). Mitochondrial swelling induced by Ca^2+^ challenge revealed that the mitochondria in D7-Des Tg mouse heart tissue were already swollen before any experimental Ca^2+^ challenges. Mitochondria from the D7-Des Tg heart also showed a significantly lower mitochondrial calcium retention capacity compared with control, indicating that the MPTP in mitochondria isolated from D7-Des Tg heart tissue were much more susceptible to Ca^2+^ loading. Mitochondrial MPTP opening caused by the increased localization of Bax to the outer membrane of the mitochondria was also evident in D7-Des Tg heart tissue. Similarly, CryAB^R120G^ Tg mouse models of DRC also showed a similar susceptibility to mitochondrial swelling induced by Ca^2^, which led to mitochondrial rupture and subsequent apoptosis (Maloyan et al., 2005). It can be speculated that protein aggregates may directly interact with mitochondria, affecting the components of either the Ca^2+^ entry pathway or the mitochondrial permeability transition pore. Indeed, studies in neurodegenerative diseases have shown that misfolded protein aggregates directly associate with mitochondrial membrane proteins resulting in mitochondrial dysfunction. Exposure of isolated mitochondria from the brain to β-amyloid resulted in the dissipation of mitochondrial membrane potential, which is a common feature of mitochondrial Ca^2+^ overload (Casley et al., 2002). Moreover, β-amyloid can also trigger MPTP opening, a result of activation of mitochondrial swelling (Cardoso et al., 2001).

**FIGURE 2:**
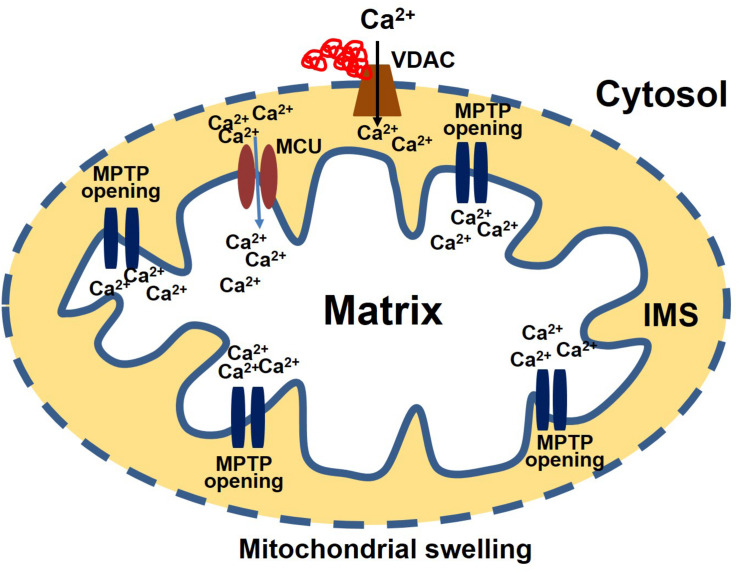
Protein aggregate interacts with VDAC leading to an increase in Ca^2+^ susceptibility. Aggregate proteins may interact with the VDAC, affecting the entry of Ca^2+^ into the mitochondria. Ca^2+^ enters the matrix via the mitochondrial calcium uniporter (MCU). After entering the matrix, Ca^2+^ binds to the mitochondrial permeability transition pore (MPTP) and triggers it to open. Excessive mitochondrial Ca^2+^ causes the MPTP to remain open for a long time. As a result, the mitochondria undergo swelling and rupture, releasing apoptosis-inducing factors into the cytosol.

Consistent with the effects observed in neurodegenerative diseases, protein aggregates in CryAB^R120G^ Tg heart tissue interact with mitochondrial protein, affecting the entry of Ca^2+^ into the mitochondria and influencing MPTP opening (Maloyan et al., 2005). Evidence for a possible interaction of the aggregate protein with the mitochondria was provided by a study showing aggregate protein accumulation in a mitochondrial fraction isolated from CryAB^R120G^ Tg heart and CryAB^R120G^ immunoprecipitation with mitochondrial protein voltage-dependent anionic channel (VDAC) (Maloyan et al., 2005). Observations of the interaction of aggregate protein CryAB^R120G^ with VDAC provides a deeper insight into mitochondrial susceptibility to Ca^2+^ overload, oxidative stress, and reduced ATP synthesis in cardiac proteotoxicity. VDAC are highly abundant proteins in the outer mitochondrial membrane (OMM). The conformational state of the VDAC depends on the voltage, with different selectivity and permeability for small ions (Rostovtseva and Colombini, 1996; Hodge and Colombini, 1997). VDAC is considered the critical channel for the exchange of metabolites and small ions between the cytosol and the mitochondrial intermembrane space (IMS) (Camara et al., 2010, 2011). Interaction of protein aggregates with the VDAC impairs VDAC activity, altering the entry of Ca^2+^ into the mitochondria (Figure 2). Interestingly, in the mouse model of DRC in which protein aggregation alters the organization of the cytoskeleton (provided by desmin and tubulin, for example) and the interaction of the aggregates with VDAC, cardiac proteotoxicity alters mitochondrial Ca^2+^ loading, leading to MPTP opening and apoptotic cell death. Studies also suggest that tubulin-mediated regulation of VDAC function limits mitochondrial metabolism and alters the IMM potential (ΔΨm), as well (Rostovtseva et al., 2008; Rostovtseva and Bezrukov, 2012). Cardiac LC amyloidosis also altered mitochondrial function in cardiac fibroblasts exposed to patient-derived pre-amyloidogenic LC (Imperlini et al., 2017) through the interaction of the amyloidogenic LC with the mitochondrial protein VDAC1 and optic atrophy protein 1 (OPA1) (Lavatelli et al., 2015).

## Mitochondrial Proteostasis in Cardiac Proteinopathy

**FIGURE 3:**
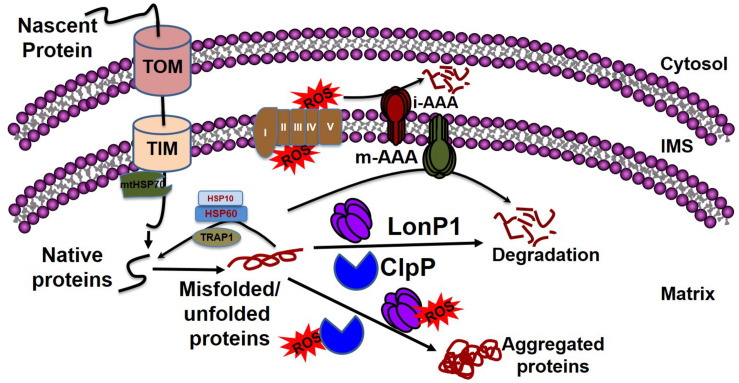
Mitochondrial quality control by chaperones and proteases. Mitochondrial proteins enter into the mitochondria through the TOM and TIM complexes with the help of HSP70, which disaggregates the protein. Following a cascade of the chaperone proteins TRAP1, HSP10, and HSP60, the protein develops its native conformation. ROS generated from the leakage of electrons from the electron transport chain can affect mitochondrial protein conformation. The inner membrane-embedded protease i-AAA degrades the misfolded proteins in the intermembrane space, while m-AAA acts on misfolded proteins in the matrix. Misfolded proteins can be refolded to their native forms by the chaperone proteins, including TRAP1, HSP60, and HSP10. The soluble proteases LonP1 and ClpP clear the aggregates in the matrix. If excessive ROS generation compromises the function of LonP1 and ClpP, the protein cannot be refolded, resulting in aggregate formation in the mitochondria leading to mitochondrial dysfunction.

Mitochondrial chaperones and proteases maintain proteostasis through the mitochondrial unfolded protein response (UPR^mt^) regulated by mitochondrial-to-nuclear communication. UPR^mt^ is a conserved transcriptional response activated by multiple forms of mitochondrial dysfunction during which the molecular chaperones function to refold the damaged proteins and misfolded proteins are degraded by proteases. Mitochondria contain approximately 1100 proteins, of which only 13 are encoded by their own genome; the rest are encoded by the nuclear genome. Nuclear encoded mitochondrial proteins are transported to the mitochondria through the mitochondrial outer and inner membrane transport machinery, which is generally termed as TOM (translocase of outer membrane) and TIM (translocase of inner membrane). For efficient transport, the transporting polypeptide needs to be in an unfolded condition; immediately after entry into the mitochondria, the unfolded polypeptide needs to be refolded. The canonical cytoplasmic proteostasis systems, including those of heat-shock proteins and proteasomes, are incapable of eliciting actions inside the mitochondria, as the mitochondria are double membrane-bound organelles (Figure 3). Moreover, as mentioned above, the mitochondrial complexes are made up of both mitochondrial genome-encoded and nuclear-encoded protein. Maintaining the balance of proteins from these two sources is a prerequisite for the proper function of the complex, as imbalance can promote protein aggregation (Voos, 2013; Moehle et al., 2019). In addition, mitochondrial proteins are constantly subjected to mitochondrial oxidative stress due to the production of the ETC by ROS. Oxidative stress may lead to the oxidation of mitochondrial proteins, such as carbonylation by reactive aldehyde and the reduction of cysteine. This results in structural changes to the protein, leading ultimately to the accumulation of non-functional toxic proteins (Hoshino et al., 2014). Altogether, the inability of the canonical cytoplasmic proteostasis system to function inside the mitochondria, the requirement for the intricate balance between nuclear and mitochondrial encoded proteins, and the vulnerability of the mitochondrial protein to oxidative stress requires the specialized mitochondrial proteostasis system as described below.

### Mitochondrial Chaperone

Accumulating evidence has implicated the disturbance of mitochondrial proteostasis in the cellular toxicities associated with neurodegenerative diseases. Only recently have researchers begun to pay more attention to understanding how the molecular mechanisms of mitochondrial proteostasis contribute to the development of cardiac proteotoxicity. Among the mitochondrial chaperones, heat shock protein 60 (HSP60) has been well studied due to its molecular function in the transportation and refolding of proteins from the cytoplasm into the mitochondrial matrix. The molecular function of HSP60 in mitochondrial protein transport is mediated by catalyzing the folding of proteins destined for the matrix and maintaining the protein in an unfolded state for transport across the inner membrane of the mitochondria (Koll et al., 1992). Studies carried out using animal models of HF have shown that the cellular distribution of HSP60 changes as a cardiac pathology evident progresses (Lin et al., 2007). In the rat coronary ligation model of cardiac injury, the cellular distribution of HSP60 was altered; increased expression was observed in the mitochondria along with a concomitant decrease in the cytosol (Lin et al., 2007). Elevated expression of HSP60 has also been reported in human hearts with dilated and ischemic cardiomyopathy (Knowlton et al., 1998). Similar to HSP60, another chaperonin heat shock protein 10 (HSP10) also participates in various aspects of HSP60 and is an essential component of the mitochondrial protein folding apparatus (Jia et al., 2011). HSP10 potential function involved in the folding and assembly of the proteins imported into the matrix compartment (Hohfeld and Hartl, 1994). Moreover, HSP60 and HSP10 have been reported to function together as a complex in the mitochondria (Figure 3) and their simultaneous overexpression confers protection against simulated ischemia (Lau et al., 1997) and ischemic injury in cardiomyocytes (Lin et al., 2001; Hollander et al., 2003). HSP 60 and HSP 10 have also shown promise as cardioprotection against doxorubicin-induced cardiomyopathy (Shan et al., 2003).

Similarly, another mitochondrial 70-kDa heat shock protein (mtHsp70, also known as Mortalin) has been reported to be involved in cardiac protein homeostasis (Shepherd et al., 2018). MtHsp70 resides in the mitochondrial matrix and is a core subunit of the presequence translocase-associated motor (PAM) complex (Figure 3). MtHsp70 has been reported to attach to the importing protein by anchoring to the translocase of the inner membrane 44 (TIM44). TIM44 helps the translocation by trapping and pulling the anchored protein through the inner mitochondrial membrane. In conjunction with the translocation of the protein, mtHsp70 also provides the proper folding of the translocating protein (Baseler et al., 2012). Proteomic analysis revealed the relevance of mtHsp70 to neurodegenerative diseases such as Parkinson’s disease (PD) (Jin et al., 2006). Decreased expression of mtHsp70 leads to compromised mitochondrial function, affecting mitochondrial protein import with concomitant decreased antioxidant defense and resulting in an increase in protein misfolding (Baseler et al., 2012). *In vitro* overexpression of mtHSP70 by adenoviral infection protects against simulated ischemia–reperfusion (IR) injury by enhancing antioxidant protein import and reducing ROS generation (Williamson et al., 2008). Moreover, it has been suggested that mtHsp70 restores mitochondrial function by decreasing changes to the mitochondrial protein structure through the inhibition of ROS generation as a result of increased import of nuclear-encoded mitochondrial antioxidant protein. Indeed, Hsp70 provides protection against myocardial injury in transgenic Hsp70 mice, presumably by refolding the misfolded protein caused by excessive ROS production (Marber et al., 1995).

Another chaperone protein localized to the mitochondria is a 75 kDa heat shock protein (HSP75 or TRAP1) known to function as a negative regulator of mitochondrial respiration owing to its ability to modulate the balance between oxidative phosphorylation and aerobic glycolysis (Yoshida et al., 2013). Studies have suggested that the impact of TRAP1 on mitochondrial respiration may be mediated by modulation of mitochondrial c-SRC and inhibition of succinate dehydrogenase (Sciacovelli et al., 2013). Mutation in TRAP1 is associated with decreased oxidative phosphorylation (OXPHOS) activity and loss of membrane potential in PD (Fitzgerald et al., 2017). Studies have demonstrated that a physiological function for TRAP1 in the heart as TRAP1 expression in the heart has been shown to decrease during natural aging (Ayyadevara et al., 2016). Overexpression of TRAP1 has been shown to protect against pressure overload-induced cardiac hypertrophy (Zhang et al., 2011). In addition, TRAP1 expression has been reported to be induced by various stresses such as oxidative injury (Hua et al., 2007; Pridgeon et al., 2007; Landriscina et al., 2010). Overexpression of TRAP1 elicits protection against cardiac hypertrophy by improving mitochondrial function, presumably by maintaining mitochondrial proteostasis. Studies have also shown that TRAP1 protects the cardiomyocyte from hypoxic injury by regulating mitochondrial permeability transition pores (Xiang et al., 2010).

### Mitochondrial Protease

In parallel with the mitochondrial chaperones, mitochondrial proteases play a pivotal role in the maintenance of mitochondrial proteostasis (Figure 3). Mitochondria contain four compartmentally based proteases: mitochondrial matrix-localized LonP1 and ClpP, and mitochondrial inner membrane ATP-dependent AAA family proteases (ATPases associated with diverse cellular activities) including i-AAA and m-AAA. Although both i-AAA and m-AAA are embedded in the inner mitochondrial membrane and contain ATPase and protease domains, their protease functions are spatially different; i-AAA is oriented in the direction of the intermembrane space, whereas m-AAA is oriented in the direction of the matrix. Due to spatial relationships, the inner membrane proteases are important for the surveillance of the OXPHOS machinery, which are constantly susceptible to oxidative damage due to their continuous production of ROS. Among these four proteases, LonP1 has been more studied for its role in the heart. LonP1 is associated with cardioprotection by ischemic preconditioning (IPS) through inhibiting ROS generation and consequent reduction in protein misfolding. Overexpression of Lonp1, as shown in LonP1 Tg mouse hearts, elicited cardioprotection by reducing the infarct size in IR injury (Venkatesh et al., 2019). Induction of LonP1 expression in cardiomyocytes has been reported in response to cellular and cardiac disturbances. Increased expression of LonP1 and ClpP1 has been reported in the hearts of Friedreich’s ataxia mice associated with the loss of mitochondrial iron–sulfur proteins during the progression of the disease (Guillon et al., 2009). In addition to unfolded or oxidized protein, LonP1 has been reported to degrade the phosphorylated proteins as indicated by degradation of phosphorylated cytochrome c oxidase under hypoxic conditions (Sepuri et al., 2017). Phosphorylation may alter the three dimensional structure of cytochrome C oxidase, affecting its function and leading ultimately to its degradation by LonP1 (Sepuri et al., 2017). Recently, it has been shown that LonP1 deficiency leads to the activation of both the endoplasmic reticulum (ER)-unfolded protein response (UPR^ER^) and the mitochondrial unfolded protein response (UPR^mt^) (Lu B. et al., 2019), resulting in a metabolic shift toward glycolysis, glycogenesis, and amino acid metabolism from the mitochondrial oxidative phosphorylation to relieve mitochondrial stress. However, the molecular mechanisms of LonP1 mediated induction of UPR^ER^, as well as UPR^mt^ and molecular signaling between UPR^ER^ and UPR^mt^, remains unknown. It is possible that UPR^ER^ acts before UPR^mt^ as a first defense to protect the mitochondria against mitochondrial stress (Lu B. et al., 2019). Moreover, UPR^ER^ induction by LonP1 deficiency could be a feedback response mechanism, as suggested by the fact that overexpression of LonP1 requires the activity of protein kinase R-like ER kinase (PERK) regulated transcription factor activating transcription factor 4 (ATF4) (Onat et al., 2019). Future studies may provide insight in this regard (Lu B. et al., 2019). Apart from the LonP1-mediated signaling during cardiac stress, it has also been suggested that oxidative modification of LonP1, such as carbonylation, tyrosine nitrosylation, and cysteine reduction, can inhibit the proteolytic activity of LonP1 in pressure overloaded mice hearts (Hoshino et al., 2014).

### Communication Between Mitochondria and the Nucleus in UPR^mt^

**FIGURE 4:**
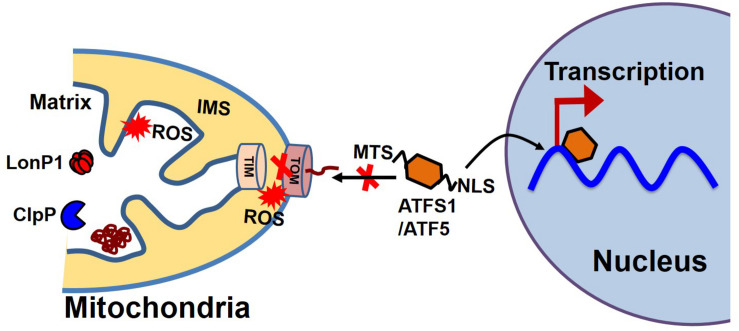
Mitochondrial and nuclear communication during UPR^mt^. Mitochondrial and nuclear communication is mediated through the transcription factor ATFS1 in *C. elegans* and ATF5 in mammals. ATFS1/ATF5 contains both a mitochondrial target sequence (MTS) and a nuclear localization signal (NLS). Under basal conditions, the mitochondrial transport system functions optimally. ATFS1/ATF5 preferentially enters the mitochondria and is degraded by mitochondrial proteases, including LonP1 and ClpP. Under stress conditions, mitochondrial entry systems are compromised due to excessive ROS generation by the mitochondria, making conditions favorable for ATFS1/ATF5 to enter the nucleus and trigger the array of gene transcription for UPR^mt^.

Most proteins involved in mitochondrial biogenesis are controlled by nuclear transcription requiring tight communication between the two organelles. Under cellular conditions requiring high mitochondrial function, coordinated transcriptional activation of both nuclear and mitochondrial genes results in an increase in mitochondrial mass (Kotiadis et al., 2014). In *C. elegans*, communication between the mitochondria and the nucleus is mediated by the activating transcription factor associated with stress (ATFS-1), which has both mitochondrial and nuclear-targeted sequences (Figure 4). ATFS-1 is routed to the mitochondria under basal conditions when mitochondrial membrane potential is high and protein import is optimal where it is degraded by mitochondrial protease. The mitochondrial target sequence of ATFS-1 has a low net charge, and cannot enter into the mitochondria with low mitochondrial membrane potential. Under this scenario, ATFS-1 is routed to the nucleus to initiate the transcription of an array of genes to mitigate mitochondrial stress (Nargund et al., 2012; Rolland et al., 2019). In mammalian mitochondria, activating transcription factor 5 (ATF5, an ortholog of ATFS1) has been reported to regulate the mitochondrial unfolded protein response (UPS^mt^) (Smyrnias et al., 2019). Studies have also shown that UPR upregulates C/EBP homologous protein (CHOP), which dimerizes with members of the CAAT/enhancer binding protein (C/EBP) family, regulating the expression of mitochondrial stress genes containing a mitochondrial unfolded protein response element (MURE) (Ryan and Hoogenraad, 2007). Another pathway of mitochondrial proteostasis regulation is mediated through a process called unfolded protein response activated by the mistargeting of proteins (UPR^am^) in yeast cells (Wang and Chen, 2015; Wrobel et al., 2015). This pathway links the defects in mitochondrial biogenesis with proteasome activity, which buffers the consequences of a physiological slowdown in mitochondrial protein import. A mitochondrial targeting sequence (MTS) is necessary for the import of most mitochondrial protein, which requires a physiological mitochondrial membrane potential. Under conditions of decreased mitochondrial membrane potential, a massive accumulation of mitochondria precursors in the cytosol occurs, which activates the proteasome and attenuates the cytosolic synthesis of proteins (Wang and Chen, 2015; Wrobel et al., 2015).

### Mitochondrial Contribution to the Degradation of Cytosolic Protein Aggregates

**FIGURE 5:**
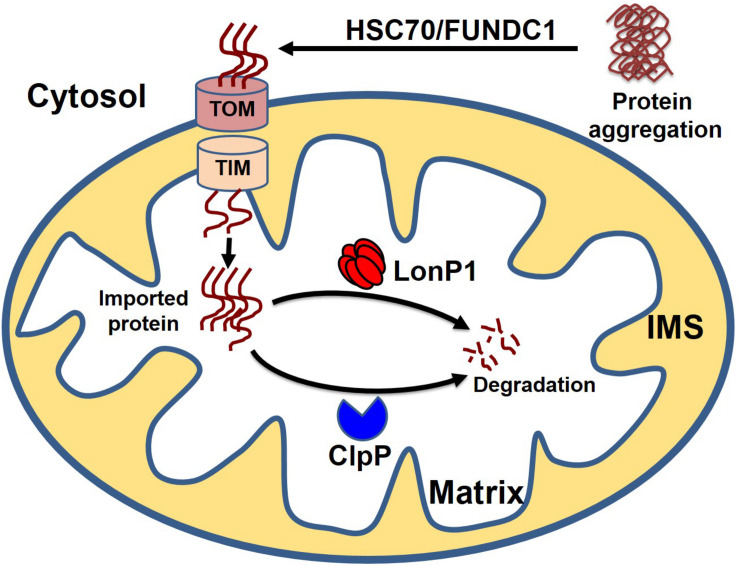
Cytosolic protein clearance by mitochondrial guidance. In addition to classical mitochondrial proteostasis, the cytosolic aggregate can be cleared by the MAGIC (mitochondria as guidance in the cytosol) pathway. Presumably, the cytosolic aggregates are disaggregated by HSC70 or FUNDC1, followed by the entry of disaggregated protein into the mitochondria. Finally, cytosolic protein transported to the mitochondria undergoes degradation by mitochondrial protease LonP1 and ClpP.

Cytosolic soluble misfolded proteins are usually degraded by the classical PQC system, including chaperones, UPS systems, and autophagy. In addition to the classical pathway, a recent study revealed another PQC pathway that involves the import of aggregate proteins into the mitochondria through mitochondrial import machinery via presumed disaggregation of the aggregate by heat shock protein 104 (HSP104). After the entry of the cytosolic aggregate into the mitochondria, the aggregates are degraded by the mitochondrial protease Pim1 (Lon protease homolog in mammals) (Ruan et al., 2017). This PQC system, which degrades the cytosolic protein imported into the mitochondria, has been given the acronym MAGIC (mitochondrion as a guardian for cytosol) (Figure 5). However, the molecular mechanism responsible for the MAGIC proteostasis pathway is not well understood and remains elusive. Extensive research is still needed to explore whether a protein without a mitochondrial targeting sequence can enter into the mitochondria, the consequence of the import of a misfolded protein on mitochondrial function, and the molecular fate of the misfolded protein in overburdened mitochondria. A recent study in an AD mouse model indicated that induction of UPR^mt^ could decrease protein aggregation, which seems to indicate that misfolded proteins from the cytosol can enter the mitochondria and ultimately become degraded (Sorrentino et al., 2017). In another study, it was shown that the downregulation of Tom40 is associated with cytosolic protein aggregation and disrupted neuronal integrity (Liu et al., 2018). Similarly, a study in human cells revealed that mitochondrial outer membrane protein FUN14 domain-containing protein 1 (FUNDC1) interacts with a 71-kDa heat shock cognate protein (HSC70) and promotes the entry of misfolded cytosolic protein into the mitochondria, followed by digestion of the translocated misfolded protein by the mitochondrial protease LonP1 (Li et al., 2019). Although it is too early to assume that the MAGIC pathway also plays a role in the heart, one could speculate that the MAGIC mechanism may also play a part in cardiac pathological stress, based on some reported evidence. Several studies have shown that decreased levels of HSP70 and mitochondrial dysfunction are positively correlated with postoperative atrial fibrillation (Mandal et al., 2005; Montaigne et al., 2013; Fauconnier, 2018). Moreover, chronic pressure overload has been associated with activation of UPR^mt^, and increased levels of mitochondrial chaperone HSP60, proteases ClpP and LonP1, and mitochondrial-nuclear communication marker ATF5 (Smyrnias et al., 2019). These studies provide some clues about the entry of misfolded cytosolic proteins into the mitochondria, followed by degradation of the aggregates by the mitochondrial protease system, indicating that the MAGIC pathway could be functionally involved in the pathological stress conditions of the heart by clearing misfolded cytosolic proteins.

## Mitochondrial Dynamics in Cardiac Proteostasis

Mitochondria are highly dynamic organelles constantly undergoing the rapid and opposing processes of fission and fusion to maintain their shape, distribution, and size. Mitochondrial fission is regulated and maintained by dynamin-related protein 1 (Drp1). Drp1 is a cytosolic protein that translocates to the outer membrane of the mitochondria where it interacts with multiple adaptor proteins, including mitochondrial fission 1 protein (Fis1), mitochondrial dynamics proteins of 49 and 51 kDa (MiD49/51), and mitochondrial fusion factor (Mff) to form fission sites where Drp1 gathers to assemble the higher-ordered spiral complexes that constrict mitochondria for asymmetric division (Loson et al., 2013; Gao et al., 2017). Mitochondrial fusion is regulated by mitofusin 1 (Mfn1) and mitofusin 2 (Mfn2) mediated fusion of the OMM, and OPA1 mediated fusion of the inner mitochondrial membrane. Physiological fission is essential for maintaining normally functioning mitochondria, but Drp1-mediated excessive fission causes mitochondrial fragmentation, mitochondrial membrane depolarization, and an increase in ROS generation and oxidative stress, all of which lead to the development of mitochondrial dysfunction (Wu et al., 2011). In fact, proteotoxic neurodegenerative diseases such as AD, Huntington disease (HD), amyotrophic lateral sclerosis (ALS), and PD exhibit excessive mitochondrial fission, producing increased levels of ROS and defective mitochondrial function (Reddy, 2014b). Moreover, biochemical experiments in affected neurons have shown that the causative mutant proteins, including β-amyloid, phosphorylated Tau, mutant Htt, mutant LRRK2, and mutant DJ1 proteins, interact with the mitochondria (Reddy, 2014a). Inhibition of excessive Drp1 activity through blocking its interaction with Fis1 showed protective effects in HD (Guo et al., 2013) and PD models (Qi et al., 2013). Studies have also demonstrated that the interactions of misfolded protein aggregates with mitochondria ultimately result in impairment of mitochondrial dynamics, dysfunction, and neuronal damage.

**FIGURE 6:**
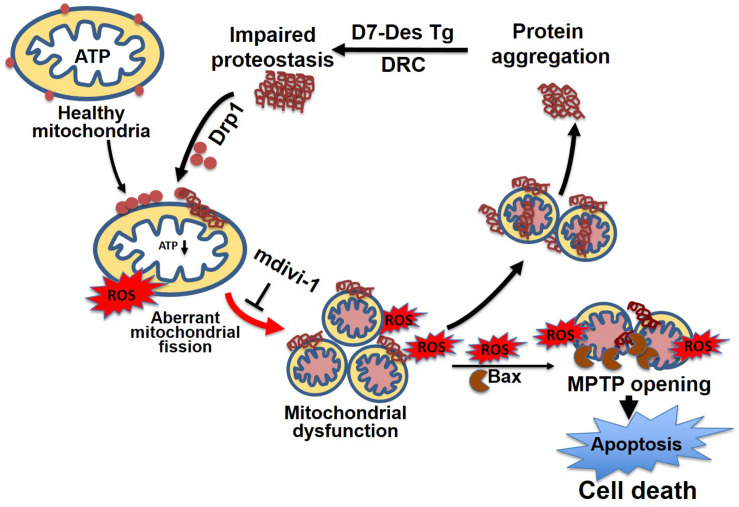
Mitochondrial dynamics in cellular proteostasis. Proteotoxicity associated with DRC leads to the accumulation of cytosolic toxic protein aggregates that activate sequential events leading to cardiomyocyte death. These cytosolic aggregate proteins activate Drp1-dependent aberrant mitochondrial fission, activate Bax-dependent MPTP opening, result in excessive ROS generation, and ultimately lead to a reduction in ATP production. The mitochondrial membrane potential is decreased as a consequence of exacerbated ROS production, which, coupled with other signaling molecules, ultimately increases the mitochondrial membrane permeability, and causes the release of apoptotic contents into the cytosol, activating apoptotic cell death. This results in decreased levels of cellular ATP, aberrant mitochondrial fragmentation, and a release of excessive ROS, which contributes to dysfunctional mitochondrial proteostasis and exacerbates the accumulation of protein aggregates. Inhibition of aberrant mitochondrial fragmentation induced by DRC in cardiomyocytes by treatment with mitochondrial fission inhibitor (mdivi-1) restored mitochondrial function and reduced the cytosolic aggregate load.

Similar to observations in neurodegenerative diseases, the DRC mice expressing either D7-Des Tg or CryAB^R120G^ Tg showed highly perturbed mitochondrial spatial organization, and myofibrils interspersed with electron-dense aggregates. In association with this, reduced mitochondrial complex I activity was observed (Reimann et al., 2003; Vincent et al., 2016). The desmin knockout mouse is reported to have defects in cardiac mitochondrial morphology, positioning, and respiratory enzyme function, demonstrating a link between mitochondrial dysfunction and desminopathy (Joshi et al., 2014). In fact, expression of DRC causing mutant proteins in cardiomyocytes is associated with aberrant mitochondrial fission and increased expression of mitochondrial fission regulatory proteins. We reported that mutated desmin expression in D7-Des Tg mouse caused a reduction in mitochondrial respiration in both isolated mitochondria and intact cardiomyocytes (Alam et al., 2018) (Figure 6). Excessive mitochondrial fission is also attributable to hypertensive cardiac hypertrophy (Hasan et al., 2018) and sepsis-induced cardiomyopathy (Haileselassie et al., 2019). Aberrant mitochondrial fission may involve several possible mechanisms such as excessive production of reactive oxygen species and activation of fission regulatory proteins (Hasan et al., 2018).

Mitochondrial fission, fusion, and mitophagy are closely related processes that combat cellular proteotoxic stress. Mitophagy has been shown to be transiently activated with concomitant translocation of Drp1 to the mitochondria during the early stages of pressure-overload HF as an adaptive stress response. However, mitophagy is downregulated during the chronic phase of HF, in conjunction with a decrease in Drp1-dependent mitochondrial fission accompanied by mitochondrial enlargement. Surprisingly, the stimulation of mitophagy by Tat-Beclin (TB1) partially rescues the cardiac dysfunction, suggesting that mitophagy activation represents a potential therapeutic target in cardiac injury (Shirakabe et al., 2016). In the heart, one of the hallmarks of aging is accumulation of the lipofuscin inclusion body, which is made up of modified protein and lipid aggregate. Mitochondrial ROS have been implicated in the formation of lipofuscin aggregate in cardiomyocytes. The mitochondria in the aging heart are also larger in size as the result of the loss of mitochondrial fission and the consequent failure of mitophagy. Moreover, inhibition of mitochondrial fission has been shown to result in increased lipofuscin formation (Terman and Brunk, 2005). Reduced mitochondrial fission accompanied by loss of mitophagy in the senescent cell has also been reported (König et al., 2017; Rizza et al., 2018). Recently, it has been shown that mice deficient in both protein kinase B (Akt) and AMP-activated protein kinase (AMPK) are predisposed to cardiac aging, presumably due to compromised autophagy and mitophagy (Wang et al., 2019). Both mitochondrial fission and fusion regulatory proteins are decreased in the aged heart; a slight concomitant increase in UPR^mt^ and mitophagy markers has been observed in mice (Song et al., 2017). Complete abolishment of both fission (Drp1) and fusion (Mfn1 and Mfn2) markers in the mouse heart caused elevation of UPR^mt^ along with decreased mitophagy, indicating that complete abolishment of mitochondrial dynamics may not activate mitophagy (Song et al., 2017). In addition to the mitochondrial dynamics, the accumulation of misfolded protein causes severe damage to the mitochondria leading to the degradation of the mitochondria by mitophagy. It has been shown that protein aggregation in the mitochondrial matrix activates the mitochondrial unfolded response as well as Pink1/Parkin-mediated mitophagy to alleviate the proteotoxicity and the mitophagy selectively facilitated by fission in mammals (Burman et al., 2017). Inhibition of mitochondrial fission results in the accumulation of large-sized mitochondria, leading to defects in mitophagy (Burman et al., 2017).

## Mitochondria-Targeted Therapy to Preserve Cardiac Proteostasis

Mitochondria-targeted therapy represents a potential strategy for the treatment of proteostasis imbalance in the heart. So far, very few studies have demonstrated any possible mitochondrial-targeted therapy for the treatment of cardiac proteinopathy or proteostasis imbalance. Several studies have provided support for the idea that the mitochondria could be a therapeutic target. Possible mitochondrial therapy could include antioxidants that prevent mitochondrial protein modification due to excessive oxidative stress, agents that help preserve mitochondrial dynamics during stress conditions, and pharmacological agents that stimulate mitochondrial stress and thereby maintain mitochondrial proteostasis. It is evident that mitochondria play a central role in maintaining both cytosolic and their own proteostasis. There is considerable cross-talk between global proteostasis and mitochondrial proteostasis. The oxidative stress experienced by the mitochondria might endanger cytosolic proteostasis, whereas the mitochondrial import of misfolded cytosolic proteins could be a possible mechanism to preserve cytosolic proteostasis.

Antioxidants targeting the mitochondria have the potential to preserve proteostasis against the pressure overload induced heart failure. A pressure overloaded HF model showed the accumulation of misfolded LonP1 due to excessive oxidative stress, and treatment with antioxidant rescued the heart failure (Hoshino et al., 2014). Moreover, in TAC mice, treatment with a mitochondria-targeted superoxide dismutase mimetic, triphenylphosphonium chloride (mito-TEMPO), decreased the level of reduced cysteine in LonP1 and restored the proteolytic activity to degrade fluorescein isothiocyanate-labeled casein (Hoshino et al., 2014). Mito-TEMPO also has been reported to prevent HF by reducing mitochondrial ROS production in both the mitochondrial and cytosolic compartments. Notably, treatment with mito-TEMPO after the onset of cardiac hypertrophy reversed cardiac remodeling, demonstrating the relevance of mitochondrial targeting/ROS scavenging as a therapy following the onset of disease (Dey et al., 2018). Another mitochondrial-targeted antioxidant is MitoQ (mitoquinone), which contains a naturally occurring antioxidant, ubiquinone, conjugated with the lipophilic cation triphenylphosphonium. The lipophilicity and positive charge allow the molecule to cross the cell membrane and enter the mitochondrial matrix to counteract mtROS (Murphy and Smith, 2007; Rossman et al., 2018). It acts by facilitating electron transfer between Complex I/II and Complex III and decreasing lipid peroxidation (Kelso et al., 2001). Recently, MitoQ has been shown to be protective in prolonged TAC-induced HF in mice by reducing H_2_O_2_ and improving mitochondrial respiration and calcium retention capacity (Ribeiro et al., 2018). Studies carried out both *in vitro* and *in vivo* have provided evidence that MitoQ represents a potential target for use clinically as a mitochondria-targeted therapy (Murphy and Smith, 2007; Smith and Murphy, 2010; Rossman et al., 2018), which has led to its inclusion in several clinical trials (Gane et al., 2010; Smith and Murphy, 2010; Snow et al., 2010). A recent clinical trial (identification no: NCT02597023) has shown that chronic supplementation with MitoQ improves vascular function in healthy older adults. These results suggest targeting mtROS using MitoQ as a potential therapeutic option for reducing age-related cardiovascular disease (CVD) (Rossman et al., 2018). Currently, MitoQ is part of a Phase 4 clinical trial to investigate the role of mitochondrial derived oxidative stress on exercise capacity and arterial hemodynamics in heart failure with preserved ejection fraction (HFpEF) patients with and without chronic kidney disease (NCT03960073, clinicaltrials.gov).

We recently reported that excessive mitochondrial fission contributes to the disease process of DRC in the mouse. Inhibition of mitochondrial fission by mdivi-1 inhibited protein aggregation and rescued mitochondrial function, including the restoration of mitochondrial oxygen consumption (Alam et al., 2018). Mdivi1 is an allosteric inhibitor of GTPase assembly and inhibits GTP hydrolysis (Cassidy-Stone et al., 2008). Mdivi-1 has also been shown to confer cytoprotection in ischemia–reperfusion injury (Ong et al., 2010) (Sharp et al., 2014) and doxorubicin-induced cardiomyopathy (Gharanei et al., 2013) by reducing the production of ROS, attenuating cytosolic calcium overload, restoring mitochondrial membrane potential, and delaying hypercontracture of cardiomyocytes. However, a recent study reported that mdivi-1 functions as a reversible inhibitor of mitochondrial complex I, affecting mitochondrial respiration on COS-7 cells (Bordt et al., 2017). Similarly, mdivi-1 impaired DNA replication and repressed mitochondrial respiration independent of Drp1 in multidrug-resistant tumor cells (Qian et al., 2014). Treatment with mdivi-1 under high glucose-induced energy stress increased complex I activity and mitochondrial density in human neuronal SK cells (Huang et al., 2015). Although extensive studies use mdivi-1 as a mitochondrial fission inhibitor, other studies published in the literature to date have demonstrated context and cell type-dependent off-target effects of mdivi-1 through Drp1 dependent/independent pathways. Another mitochondrial fission inhibitor is P110, a 7-amino acid peptide harboring a homolog sequence between Fis1 and Drp1. This peptide selectively inhibits the interaction between Drp1 and Fis1. Several growing bodies of evidence indicate that P110 mediates mitochondrial fission inhibition and rescues the mitochondrial structure and interconnectivity in proteotoxic neuronal animal models of ALS (Joshi et al., 2018a), and Parkinson disease (Filichia et al., 2016). P110 treatment showed a reduction in Aβ accumulation, energetic failure, and oxidative stress in the AD mouse model (Joshi et al., 2018b). P110 also has been shown to play a protective role in the heart subjected to the transient coronary artery occlusion (Ong et al., 2010). The mechanism underlying the protective effect of fission inhibition under stress conditions is presumably that it controls ROS generation, thereby protecting the protein against ROS induced modification.

Several studies have suggested that the UPR^mt^ and mitophagy pathways can be effectively induced in various mammalian tissues by supplementation with NAD^+^-boosting compounds, including nicotinamide riboside (NR) and olaparib (AZD2281 or AZD) (Mouchiroud et al., 2013; Fang et al., 2016; Gariani et al., 2016; Zhang et al., 2016). Increasing the levels of NAD^+^ by supplementation with NR induces the UPR^mt^ in cardiomyocytes and is implicated in the protective effect against chronic pressure overload in the mouse heart (Smyrnias et al., 2019). The beneficial effects of boosting NAD^+^ were also observed in agonist-induced pathological hypertrophy, chronic pressure overload, and mitochondrial cardiomyopathy associated with Friedreich’s ataxia (Pillai et al., 2010; Lee et al., 2016). A recent clinical study elucidated the pharmacokinetics of NR in healthy volunteers and reported that NR is safe in humans (Airhart et al., 2017). Several current clinical trials are investigating the efficacy of boosting NAD + to improve mitochondrial function in treating cardiomyopathy (NCT03727646, NCT03423342 clinicaltrials.gov) and Friedreich’s ataxia (NCT04192136 clinicaltrials.gov).

## Outlook and Conclusion

Mitochondria are essential organelles that play numerous critical metabolic roles in cardiomyocytes, and their dysfunction leads to the development of different cardiovascular diseases. Despite extensive studies of the biology of the mitochondria with respect to health and disease, the molecular processes involved in mitochondrial proteostasis remain relatively unstudied in the heart. Most of the proteotoxic diseases, including cardiac proteotoxicity and neurodegenerative diseases, are associated with the accumulation of misfolded protein aggregates, which leads to the progressive development of cellular pathology as organisms age. Importantly, in animal models of proteotoxicity, such as DRC, PD, and AD mouse models, mitochondrial dysfunction develops before the clinical manifestation of the disease. These studies suggest the possible involvement of dysfunctional proteostasis at the early stages of disease, leading to the gradual overwhelming of the mitochondrial PQC, alteration of mitochondrial dynamics (biogenesis as well as function), and eventual alteration of mitophagy, which may explain the gradual development of proteotoxic diseases during aging. Therefore, understanding the molecular signaling regulating mitochondrial proteostasis and its dysfunction under pathological conditions is critical to developing new therapies. Studies suggest that the mitochondria may contribute to clearing the cytosolic aggregate in non-cardiac cells (Ruan et al., 2017; Sorrentino et al., 2017), and there is a possibility that the mitochondria in cardiac cells may also take up cytosolic aggregates (Fauconnier, 2018). In this context, studies with direct molecular evidence showing the participation of cardiac mitochondria in the clearance of cytosolic protein aggregates remain unknown. Therefore, future studies are required to determine whether the cardiac mitochondria function to clear the cytosolic protein aggregates and, if so, to dissect the molecular mechanism. Research over the past few decades has identified multiple mechanisms and potential therapeutic targets, especially mitochondrial-targeted therapy, for the protection of cardiac proteostasis. Moving these targets into therapy requires recognizing the multitude of mitochondrial mechanisms in cardiac proteostasis. Future investigations of the balance among mitochondrial function regulators, including ROS, Ca^2+^, and the redox state, would minimize the gap. In this review, we have gathered together the current understanding of the molecular signaling and regulatory mechanisms of mitochondrial proteostasis under pathophysiological conditions. However, to develop new therapies, further studies are required to understand the deeper mechanisms of mitochondrial proteostasis in cardiac diseases.

## Author Contributions

SA and MB conceptualized, designed, and wrote the manuscript. CA, RA, and MM participated in the conceptualization and editing of the manuscript. All co-authors edited and proofread the manuscript and approved the final version.

## Conflict of Interest

The authors declare that the research was conducted in the absence of any commercial or financial relationships that could be construed as a potential conflict of interest. The reviewer JS declared a past co-authorship with one of the authors with several of the authors MB, SA, and RA to the handling Editor.

## Abbreviations

Δψ: mitochondrial membrane potential
ΔΨ m: IMM potential
AAA: ATPases associated with diverse cellular activities
AD: Alzheimer’s disease
Akt: protein kinase B
ALS: amyotrophic lateral sclerosis
AMPK: AMP-activated protein kinase
ATF4: activating transcription factor 4
ATF5: activating transcription factor 5
ATFS-1: activating transcription factor associated with stress
Ca^2+^: calcium
CHOP: C/EBP homologous protein
ClpP: caseinolytic mitochondrial matrix protease proteolytic subunit
COA6: cytochrome c oxidase assembly factor 6 homolog
COX10: cytochrome C oxidase assembly homolog 10
COX15: cytochrome C oxidase assembly homolog 15
COX2: cytochrome C oxidase subunit 2
CryAB: α B-crystallin
CryAB^R120G^: cardiomyocyte-specific overexpression of mutant (R120G) CryAB
D7 − Des Tg: desmin transgenic mouse with 7-amino acid deletion R172 − E178 in desmin
DRC: desmin-related cardiomyopathy
Drp1: dynamin-related protein 1
ETC: electron transport chain
Fis1: mitochondrial fission 1 protein
FUNDC1: FUN14 domain-containing protein 1
H_2_O_2_: hydrogen per oxide
HD: Huntington disease
HDAC6: histone deacetylase 6
HF: heart failure
HSC70: heat shock cognate 71-kDa protein
HSP104: heat shock protein 104
HSP60: heat shock protein 60
HSP70: heat shock protein 70
HSP75/TRAP1: heat shock protein 75 kDa
iCa^2+^: intracellular calcium
IF: intermediate filament
IMF: interfibrillar mitochondria
IMS: mitochondrial intermembrane space
IPS: ischemic preconditioning
IR: ischemic-reperfusion
LC: light chain
LonP1: Lon protease I
MAGIC: mitochondrion as a guardian for cytosol
mCa^2+^: mitochondrial calcium
MCU: mitochondria calcium uniporter
Mff: mitochondrial fusion factor
Mfn1: Mitofusins 1
Mfn2: Mitofusins 2
MiD49/51: mitochondrial dynamics proteins of 49 and 51 kDa
Miro2: mitochondrial Rho GTPase 2
MitoQ: mitoquinone
mito-TEMPO: triphenylphosphonium chloride
MnSOD: manganese (Mn)-dependent superoxide dismutase
MNTs: membrane nanotubes
MPTP: mitochondrial permeability transition pore
MT: Microtubulin
mtDNA: mitochondrial DNA
mtHsp70: mitochondrial 70-kDa heat shock protein
MURE: mitochondrial unfolded protein response element
NCLX: mitochondrial sodium-calcium exchanger
OMA1: metalloendopeptidase OMA1
OMM: outer mitochondrial membrane
OPA1: optic atrophy protein 1
OXPHOS: oxidative phosphorylation
PD: Parkinson disease
PERK: protein kinase R-like ER kinase
Pim1: Lon protease homolog in mammals
PNM: perinuclear mitochondria
PolG: mitochondrial polymerase gamma
PQC: protein quality control
RNOS: reactive nitrogen and oxygen species
ROS: reactive oxygen species
SCO1: synthesis of cytochrome oxidase1
SCO2: synthesis of cytochrome oxidase2
SDH: succinate dehydrogenase
SSM: subsarcolemmal mitochondria
TAC: transverse aortic constriction
TIM: translocase of inner membrane
TIM44: translocase of inner membrane 44
TOM: translocase of outer membrane
Tom40: translocase of outer membrane 40
UPR: unfolded protein response
UPR^am^: unfolded protein response activated by the mistargeting of proteins
UPR^ER^: endoplasmic reticulum (ER)-unfolded protein response
UPR^mt^: mitochondrial unfolded protein response
UPS^mt^: mitochondrial unfolded protein response
VDAC: voltage-dependent anionic channel.
